# Determining post-orthognathic surgery profile: A validation study on the potential of modified chin point as a reliable predictor

**DOI:** 10.1371/journal.pone.0329535

**Published:** 2025-08-22

**Authors:** Pradeep Singh, Huijun Han, Heng Zhang, Richard Tai‑Chiu Hsung, Yifan Lin, Deepal Haresh Ajmera, Yiu Yan Leung, Lifeng Zhu, Congyi Zhang, Min Gu

**Affiliations:** 1 Discipline of Orthodontics, Faculty of Dentistry, The University of Hong Kong, Hong Kong SAR, China; 2 Department of Computer Science and Engineering, Texas A&M University, College Station, Texas, United States of America; 3 Department of Computer Science, Hong Kong Chu Hai College, Hong Kong SAR, China; 4 Discipline of Oral and Maxillofacial Surgery, Faculty of Dentistry, The University of Hong Kong, Hong Kong SAR, China; 5 Discipline of Applied Oral Sciences and Community Dental Care, Faculty of Dentistry, The University of Hong Kong, Hong Kong SAR, China; 6 State Key Laboratory of Digital Medical Engineering, School of Instrument Science and Engineering, Southeast University, Nanjing, China; 7 Department of Computer Science, Faculty of Engineering, The University of Hong Kong, Hong Kong SAR, China; Hospital São Lucas da PUCRS: Hospital Sao Lucas da PUCRS, BRAZIL

## Abstract

**Objectives:**

To investigate the validity of the modified chin point (MCP) as a reliable predictor for post-orthognathic surgery profile.

**Materials and methods:**

136 three-dimensional (3D) facial images from 68 patients (28 males and 40 females; mean age 24.6 ± 5.3 years) were collected. An artificial intelligence (AI)-assisted in-house software program was developed to automatically localize landmarks, compute the MCP, and perform automated distance measurements. The Steiner’s *S* line and Ricketts’ *E* line were computed for each scan to assess lip positioning relative to the reference lines. Validity was established by comparing MCP measurements to those from the actual chin point (ACP).

**Results:**

No significant differences (p > 0.01) were observed in upper lip (UL) and lower lip (LL) positions between post-surgery ACP and MCP. Mean error (ME) and mean absolute error (MAE) for UL and LL positions in relation to ‘S’ and ‘E’ lines were generally small. Strong positive correlations were observed between ACP and MCP variables for UL measurements, while moderate positive correlations were observed for LL measurements for both ‘S’ and ‘E’ lines. Additionally, MCP-based post-surgery aesthetic lip positions did not differ significantly (p > 0.01) across genders.

**Conclusions:**

This study provides evidence supporting MCP’s effectiveness in guiding aesthetic lip positioning in patients undergoing orthognathic surgery (OS). MCP is reliable and consistent in estimating post-surgery UL position and moderately reliable in estimating post-surgery LL position, validating its use as a predictor for the post-orthognathic surgery profile.

**Clinical significance:**

The MCP-based prediction model can be easily incorporated into pre-surgical planning, allowing orthognathic surgeons and orthodontists to make appropriate adjustments to treatment plans, ensuring optimal treatment outcomes.

## Introduction

Facial attractiveness can significantly influence a person’s psychosocial wellbeing and interpersonal relationships [1–3]. Research indicates that individuals with attractive facial features are often perceived as having more desirable personality traits and are generally more likeable [4]. Among the various facial features, the lips and chin are the most noticeable and play a crucial role in determining facial beauty [5,6]. Given these findings, it is not surprising that an increasing number of patients are visiting orthognathic-orthodontic offices with a desire to enhance their facial and smile aesthetics. This desire serves as a primary motivating factor and has become one of the most common reasons for seeking orthognathic-orthodontic treatment, the foremost goal of which is to achieve a harmonious and attractive profile [7–11].

In contemporary orthognathic surgery (OS) planning, the chin is a critical element that contributes to the overall appearance of the facial profile [12,13]. It is commonly regarded as a prominent feature in the profile structure, as evident from before and after pictures on plastic surgeons’ websites or in professional articles [14]. The perceived adequacy of chin projection is dependent on its relationship with other facial features. Several aesthetic parameters contribute to the perceived attractiveness of the chin region, including the proportion of chin height relative to lower facial height, labiomental fold contour, the projection of the soft tissue chin, and its correlation with the position of the lips [13]. The position of the chin significantly affects the aesthetics of the lower face [15,16] as well as overall facial profile [17], making the reestablishment of chin morphology a crucial component of corrective jaw surgery. The chin point, or pogonion, is often used for quick assessment of the profile and the lip position during orthognathic surgery planning. To facilitate this assessment, several clinical parameters have been established, including analytical lines such as the Riedel line/Facial harmony line [18], the *Zero meridian* line [19], Ricketts’ *E* line [20], Steiner’s *S* line [21] and cephalometric angles like the Merrifield *Z-angle/Z-line* [22]. However, each of these parameters has its own inherent limitations. The Riedel line may not provide an accurate assessment of chin position in cases of lip incompetence or if the position of the pogonion doesn’t coincide with the line. Although the pogonion should lie on or immediately posterior to the zero meridian line in aesthetically pleasing profiles, it may not always hold true if the Frankfurt horizontal plane (FHP) is not estimated precisely or may vary depending on the nasion position. Likewise, Merrifield Z-angle relies on FHP, and its applicability may be influenced by the position of the pogonion. Therefore, it might offer a limited understanding of the non-procumbent lip, chin projection, and overall facial profile. Additionally, even the most consistent reference lines for lip positioning assessment- ‘S’ and ‘E’ should only be used when the chin is in a normal skeletal Class I relation and may not be applicable to patients with Class II and III positions [23]. In our previous study [23], we analyzed perceptions of facial attractiveness among dental students and laypeople in a Chinese population and found that lip positions within approximately ±2 mm of the S or E lines were consistently rated as most aesthetic. However, because these reference lines are only valid for Class I skeletal relationships, their clinical utility is limited in patients with Class II or III malocclusions. This limitation motivated the development of the modified chin point (MCP) in the present study, which allows S and E lines to be applied universally across skeletal classes. By standardizing the chin position using MCP, we aimed to extend the validated ±2 mm aesthetic criterion to all patients, regardless of skeletal classification.

To precisely evaluate the position of lips, Arnett et al. proposed altering the position of the chin point, by moving the chin point up or down, forward or backward, or left or right, to simulate the effect of orthognathic surgery or other corrective procedures followed by evaluating the position of the lips based on the new position of the chin point, thus allowing for a more accurate diagnosis and treatment plan [24,25]. However, on what grounds should the chin position be modified or altered is yet to be investigated. Moreover, the validity of the modified chin position still needs to be analysed.

In this study, we introduce the actual chin point (ACP)—defined as the most anterior point on the soft tissue contour of the chin (pogonion) in the midsagittal plane—and propose a MCP derived from the consistent angle of facial convexity observed in aesthetically balanced Chinese profiles (Table 1). The technical derivation of MCP is detailed in the methods section. In our previous work [23], we demonstrated that the “angle of facial convexity” (∠G′- Sn′- Pog′; Table 1) [26,27] in Chinese faces with aesthetic appeal remains highly consistent, with minimal variation between males and females. This consistency forms the basis for standardizing chin position using MCP.

**Table 1:** Description of the landmarks and reference lines.

Annotation	Landmark	Description	Reference
**Col**	Columella point	The most anterior point on the columella of the nose	*Aljabaa et al.* [3,1]
**Pog′**	Soft tissue Pogonion	The most prominent point on the soft tissue contour of the chin	*Athanasiou* [32]
**Pn′**	Pronasale	The most prominent point of the nose	*Athanasiou* [3,2]
**G′**	Soft tissue Glabella	The most anterior projection of the lower forehead	*Resnick et al.* [27]
**Sn′**	Subnasale	The point where the lower border of the nose meets the outer contour of the upper lip	*Athanasiou* [3,2]
**ACP**	Actual chin point	The most anterior point on the soft tissue contour of the chin in the midsagittal plane	
**MCP**	Modified chin point	A chin point obtained by horizontally projecting the ACP onto a line originating at subnasale and forming a consistent 170º angle of facial convexity with the glabella-subnasale line	
**UL**	Upper lip	The most anterior point on the upper lip	
**LL**	Lower lip	The most anterior point on the lower lip	
**Reference lines and angle**			
**S line**		A line drawn from Col to Pog′	*Steiner et al*. [21]
**E line**		A line drawn from Pn′ to Pog′	*Ricketts et al.* [20]
∠**G′- Sn′- Pog′**		Angle of facial convexity	*Anic-Milosevic et al.* [26]

In recent years, the integration of Artificial Intelligence (AI)-assisted methodologies has shown tremendous potential in various medical fields, including orthognathic surgery [28–30]. These approaches utilize advanced algorithms and automation to improve the accuracy and reliability of predicting post-surgery profiles, providing valuable insights for treatment planning. However, their application in assessing the clinical applicability of the MCP as a reliable predictor for post-orthognathic surgery profiles remains largely unexplored. By leveraging AI-assisted methodologies, it is possible to enhance the precision of profile predictions and gain a deeper understanding of the relationship between the MCP and post-surgery lip positions. Therefore, with this intent in mind, the current study aimed to investigate the validity of the modified chin point as a reliable predictor for post-orthognathic surgery profile. The objectives of the study were twofold: (1) to compare the positioning of the lips relative to the ACP with the positioning of the lips related to the MCP in OS patients; and (2) to establish post-surgery aesthetic lip position based on the MCP. The null hypothesis posited that there would be no discernible disparity between the lip positions relative to the ACP and the lip positions related to the MCP. To our knowledge, this is the first study investigating the clinical applicability of the MCP in assessing post-orthognathic surgery profiles.

## Materials and methods

### Study design

This retrospective experimental study sought to validate the feasibility of utilising a MCP in assessing the post-orthognathic surgery profile. To accomplish this, the positioning of the lips relative to the ACP in the three-dimensional (3D) facial images of OS patients was compared with the positioning of the lips related to the MCP in the 3D facial images of the same patients.

### Ethics approval and consent to participate

Prior to the commencement of the study, ethics approval (approval number UW 21-529) was obtained from the Human Research Ethics Committee (HREC) of the University of Hong Kong, Hospital Authority, Hong Kong West Cluster. The study was conducted in accordance with the protocol, standards of good clinical practice, and ethical principles specified in the Declaration of Helsinki for medical research involving human participants. Given the retrospective design of the study, informed consent from individual patients was not deemed necessary as the research involved the analysis of existing de-identified data and medical records. The waiver of informed consent for participation was approved by the HREC.

### Consent for publication

Written informed consent for publication was obtained from the patient(s) whose images are included in this manuscript. All images have been masked to protect patient identity.

### Sampling and sample

A total of 165 patient records from the orthodontic-orthognathic patient pool of the Prince Philip Dental Hospital, University of Hong Kong, who received orthognathic surgery at the same hospital between October 2000 and December 2021 were initially screened retrospectively. The data for this study, accessed by the authors for research purposes, was collected between January 2022 and April 2022. The subjects were selected if they fulfilled the following inclusion criteria: (1) Chinese subjects (similar ethnicity); (2) age > 16 years; (3) received bimaxillary surgery or mandibular corrective surgery, with or without genioplasty; and (4) had pre- (*T*_*0*_) and post-surgical (*T*_*1*_) 3D facial images. Therefore, the final sample for the present study comprised 68 patients (28 males and 40 females) with a mean age of 24.6 ± 5.3 years (age range 16–42 years). Subjects with clinically apparent facial asymmetry (soft tissue chin deviation > 3 mm), facial deformities resulting from maxillo-facial trauma or temporo-mandibular dysfunction, a history of craniofacial syndromes or craniofacial surgery, or ambiguous 3D facial images were excluded. The authors did not have access to information that could identify individual participants during or after data collection in this retrospective study. Patient records were anonymized and de-identified during the data collection process to ensure patient confidentiality and privacy.

Based on a previous study and using G*Power (version 3.1.9.6, Kiel University, Germany) to determine a clinically significant mean difference of at least 0.5 mm (standard deviation of 0.2) in measurements pre- and post-surgery, with a power of 80% and a significance level of 5% (two-sided), a minimum sample of 34 participants was required [33]. To account for potential challenges such as the non-availability of patient records or indistinct 3D facial images during the screening process, a total of 68 participants were deemed adequate for inclusion in the study.

### Data collection and pre-processing

**Fig 1:**
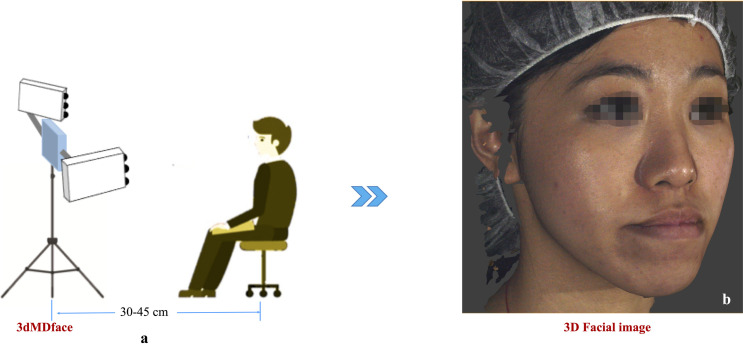
Schematic representation of the facial image acquisition using 3dMDface: (a) Imaging set-up; (b) Three-dimensional facial image generated using 3dMDface.

**Fig 2:**
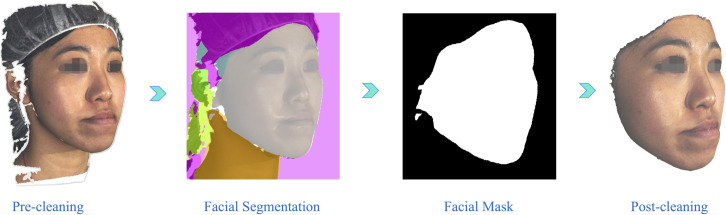
Visualization of the data cleaning operation process for 3D facial images.

A total of 136 pre- and post-surgery 3D facial images of 68 patients were collected from the orthodontic-orthognathic patient pool. Post-surgery face scans were captured at least 3 months following surgery to ensure any post-operative swelling had completely subsided. All the 3D face scans were acquired in high-definition (HD) mode using the *3dMDface* system (3dMD LLC, Atlanta, GA, USA; https://3dmd.com/) under standardised clinical imaging conditions (Fig 1). This entailed seating the participants comfortably in an adjustable chair, maintaining a distance of 30–45 cm from the imaging device, and ensuring the room had 10,000 lux and 4100 K illuminance with no windows. Each participant was advised to sit upright, adopt a natural head position (NHP) [34], with eyes wide open, and maintain minimal facial expression and maximum intercuspal position (MIP) during scanning. Next, a data cleaning operation was performed (Fig 2) to focus only on the scanned facial region and exclude any non-facial confounding variables, including hair or accessories such as head-caps, hats, or necklaces. To eliminate non-facial parts, the collected facial scans were initially rendered from three different viewpoints: the frontal view and the oblique view (right and left 45º view). Subsequently, a bilateral segmentation network called *BiSeNet* [35] was employed to extract the facial regions from the rendered multi-angle images and retain only the relevant regions for further analysis.

### Automatic landmark annotation

**Fig 3:**
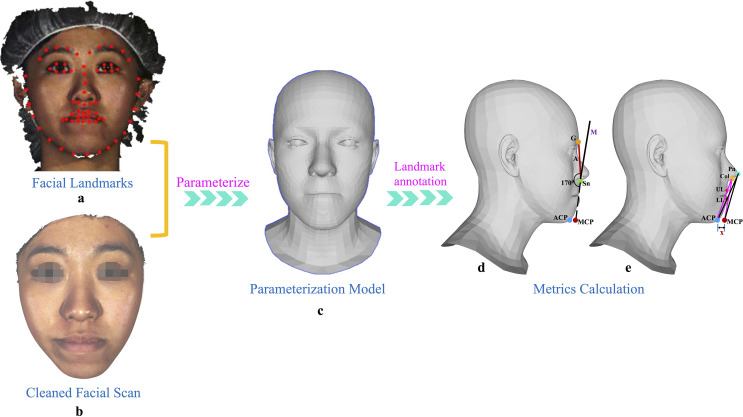
Workflow of automatic landmark annotation, modified chin determination, and metrics calculation. The ACP and MCP are represented by blue and dark red dots, respectively, in panel d and e. ‘Line A’ is depicted by a red line, while ‘Line M’ with its dashed extension is represented by a black line in panel d. The glabella (G’) and subnasale (Sn’) are represented by amber and green dots, respectively, in panel d. The pronasale (Pn’) and columella (Col) are represented by turquoise and amber dots, respectively, in panel e. The bright red dots denote the UL and LL. The horizontal distance between ACP and MCP is denoted by ‘x’ in panel e.

To facilitate the process of automatic landmark annotation, the initial step involved rendering each 3D facial scan into a two-dimensional (2D) frontal view image of the face. Next, dense connections were established between the vertices of the 3D scans and their corresponding pixels on the rendered 2D frontal view images. Subsequently, an in-house-developed program that integrates an image-based landmark detector, previously trained on a substantial dataset by Dong et al. [36,37], was employed to autonomously detect and locate the 2D coordinates of 68 landmark points (previously defined in the literature) [38] on the frontal view image (Fig 3a). The model was not trained on our cohort; instead, we utilized the publicly available pre-trained weights provided by the developers, as described in Dong et al. [37]. In order to acquire the landmarks in 3D coordinates, the preceding mapping from 3D vertices to their corresponding 2D pixels was utilized. Additionally, a ‘*Supervision by Registration’* (Fig 3a and 3b) approach was employed to further enhance the precision of the image-based landmark detector, as detailed in the referenced study [37]. Furthermore, the 3D facial scans were parameterized (Fig 3c), which enables easy selection and manipulation of the landmarks on the 3D face scans. Manual adjustments were made to correct any inconsistencies, such as drifted landmarks, in the automatic landmark annotation, particularly for patients with challenging preoperative conditions.

### Modified chin determination and measurements (Metrics calculation)

The in-house-developed program incorporates a robust algorithm that enables automatic localization of landmarks, computation of MCP, and automated distance measurement. The first step involved computing the MCP. Our prior investigation revealed a highly consistent mean ‘angle of facial convexity’ of 170° in aesthetic Chinese faces, with a mean of 170.3° ± 3.2 for males and 170.1° ± 3.2 for females. Consequently, this angle was employed as a criterion for establishing MCP (Fig 3d). To accomplish this, the algorithm employed localization techniques to identify the landmarks on the parameterized model, namely the ‘glabella’ and ‘subnasale’ (Table 1), which are associated with the ‘angle of facial convexity’. It then generated a virtual line denoted as ‘line A’ (Fig 3d), intersecting through the soft tissue landmarks ‘glabella’ and ‘subnasale’. Subsequently, ‘line M’ was calculated that commenced from ‘subnasale’ and established a 170° angle with the line A (Fig 3d). The ‘line M’ was extended vertically downwards until it reached the adjacent original pogonion position (ACP). The MCP was derived by horizontally translating the original pogonion position (ACP) onto line M. This newly defined pogonion point, adjacent to the original pogonion position (ACP) and based on the ‘angle of facial convexity’, was referred to as the “modified chin point” (Fig 3d). In summary, the MCP was defined as a chin point obtained by horizontally projecting the ACP onto a line originating at subnasale and forming a consistent 170° angle of facial convexity with the glabella-subnasale line. This approach standardizes chin positioning based on the angle of facial convexity. The MCP was determined for each pre- and post-surgery facial scan.

The S-line (Steiner’s line) was defined as a straight line drawn from the midpoint of the columella [31] (Col) to the soft tissue pogonion [32] (Pog′), and the E-line (Ricketts’ line) was defined as a line drawn from the pronasale (Pn′) to the soft tissue pogonion (Pog′). These definitions are also provided in Table 1 for reference. Following this, the algorithm identified the relevant landmarks (Table 1) on each parameterized model, and created corresponding reference lines (Fig 3e). The ‘S’ and ‘E’ lines were computed simultaneously but separately, based on the ACP and the newly determined MCP. Subsequently, the program autonomously computed the positioning of the lips, including the distance of the upper and lower lips relative to each reference line, for both pre- and post-surgery parameterized models. Furthermore, separate estimations were made for the horizontal distances (denoted by ‘x’ in Fig 3e) between the pre-surgery ACP and MCP, as well as between the post-surgery ACP and MCP (Fig 3e). The entire procedure, from landmark localization to MCP determination to the metrics computation, was seamless and automated.

### Outcome measures

**Fig 4:**
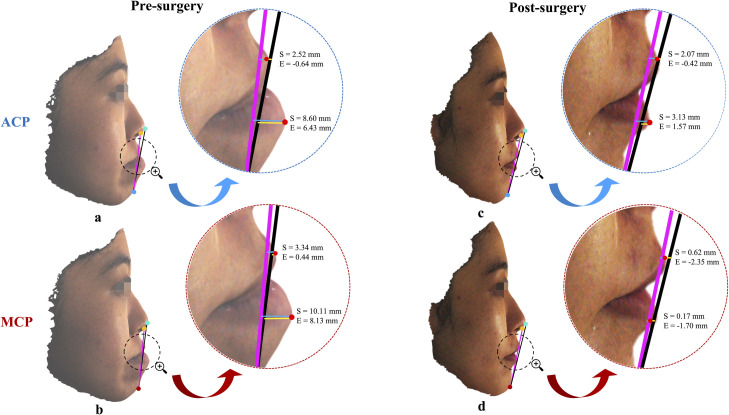
Comparison of pre- and post-surgery lip positioning relative to ACP (4a and 4c) and MCP (4b and 4d). The ‘S’ line is represented by a pink line and the ‘E’ line is represented by a black line. The turquoise, amber, blue, and dark red dots represent the pronasale (Pn′), columella (Col), ACP, and MCP, respectively, while the bright red dots represent the UL and LL. Note that the ‘S’ and ‘E’ line values are specific to this patient.

The outcome measures for the present study focused on evaluating the accuracy and applicability of the MCP in assessing lip positioning following surgery. Therefore, to quantitatively analyse the measures, the pre- and post-surgery positioning of the lips relative to the ACP were compared with the pre- and post-surgery positioning of the lips related to the MCP (Fig 4). Additionally, the horizontal distances between the pre-surgery ACP and MCP as well as between post-surgery ACP and MCP were estimated independently.

### Error study

All the measurements in this study were acquired by an automated process utilizing an in-house-developed program. Prior to data collection, the program algorithm was validated to ascertain the accuracy and reliability of the measurements [36]. The automated measurements consistently yielded the same values, thus obviating the need for an error study.

### Statistical analysis

The collected data was analysed using IBM Statistical Package for the Social Sciences (SPSS) version 25.0 (SPSS for Mac, IBM Corp., Armonk, N.Y., USA). The normality of the data distribution was validated using the *Shapiro-Wilk test*. To determine and predict post-orthognathic surgery profiles based on the MCP, various statistical tests were employed. Firstly, a *paired t-test* for dependent samples was used to assess the pre- and post-surgery positioning of the lips relative to the ACP and MCP. Additionally, a *Wilcoxon ranked sum test* was conducted to evaluate the difference in chin positions before and after surgery relative to ACP and MCP. The validity of the MCP as a predictor was assessed through the analysis of mean error (ME) and mean absolute error (MAE), as well as *Pearson correlation analysis*. ME and MAE were used to measure the average discrepancy between the predicted values based on the MCP and the actual values based on the ACP post-surgery, thus determining the accuracy and reliability of the MCP in establishing the post-surgery aesthetic lip position. *Pearson correlation analysis* was used to visually depict the relationship between ACP and MCP. Descriptive statistics, including mean, standard deviation (SD), and 95% confidence interval (CI), were computed for both upper lip (UL) and lower lip (LL) for each reference line and in relation to each gender to determine MCP based post-surgery aesthetic lip positions. Furthermore, an *independent sample t-test* was conducted to compare post-surgery aesthetic lip positions between genders. To decrease the possibility of erroneous rejection of the null hypotheses, the statistical imbalance resulting from multiple comparisons was mitigated through the use of the Bonferroni adjustment (p < 0.05/number of tests). A significance level of p < 0.01 (0.05/4) was deemed to be statistically significant.

## Results

Table 2 displays a comparative analysis of upper and lower lip positions in pre- and post-surgery groups. The measurements of the UL and LL were found to be significantly higher (*p* < 0.01) when assessed relative to the pre-surgery MCP as compared to the pre-surgery ACP for both the ‘S’ and ‘E’ lines. Conversely, there were no statistically significant differences (*p* > 0.01) in the positions of the UL and LL between post-surgery ACP and post-surgery MCP.

**Table 2:** Comparison of upper and lower Lip positions in pre- and post-surgery groups.

	*T*_*0*_							*T*_*1*_						
	ACP			MCP				ACP			MCP			
Variables	Mean ± SD (mm)	95% CI		Mean ± SD (mm)	95% CI		p*	Mean ± SD (mm)	95% CI		Mean ± SD (mm)	95% CI		p*
**UL**		Lower	Upper		Lower	Upper			Lower	Upper		Lower	Upper	
**S Line**	0.96 ± 2.78	0.29	1.63	2.33 ± 2.00	1.84	2.81	all *p* values < 0.01	1.05 ± 1.72	0.63	1.47	1.09 ± 1.71	0.68	1.51	all *p* values > 0.01
**E Line**	−2.87 ± 3.41	−3.70	−2.05	−1.04 ± 2.21	−1.58	−0.51		−2.21 ± 2.05	−2.71	−1.71	−2.15 ± 1.98	−2.63	−1.67	
**LL**														
**S Line**	6.02 ± 2.80	5.34	6.70	8.66 ± 4.52	7.56	9.75		2.07 ± 1.94	1.60	2.54	2.17 ± 2.79	1.49	2.84	
**E Line**	3.54 ± 3.12	2.78	4.29	6.49 ± 4.52	5.40	7.59		0.01 ± 2.09	−0.49	0.52	0.13 ± 2.90	−0.57	0.83	

T_0_, Pre-surgery; T_1_, Post-surgery; ACP, Actual chin position; MCP, Modified chin point; UL, Upper Lip; LL, Lower Lip; mm, millimetres; SD, standard deviation; CI; Confidence Interval; *p < 0.01, considered statistically significant

Additionally, Table 3 presents a comparison of differences in chin positions before and after surgery relative to ACP and MCP. The results demonstrated that the pre-surgery group had a significantly higher ACP-to-MCP distance (*p* < 0.01) compared to the post-surgery group. Specifically, the median distance was 7.98 mm in the pre-surgery group [ACP (*T*_*0*_) to MCP (*T*_*0*_)] and 3.83 mm in the post-surgery group [ACP (*T*_*1*_) to MCP (*T*_*1*_)] with an interquartile range (IQR) of 3.56 to 11.35 and 1.61 to 5.72, respectively, indicating the impact of orthognathic surgery on the positioning of the lips relative to the chin. Overall, the findings from Tables 2 and 3 indicate that the MCP may be a valuable tool in assessing lip position relative to chin in OS patients.

**Table 3:** Comparison of difference in chin positions between pre- and post-surgery groups.

	*T*_*0*_ *-T*_*1*_			
	Mean ± SD	Median	IQR (25^th^ – 75^th^)	p*
**Distance (mm)**				
**ACP (*T***_***0***_ **)-to-MCP (*T***_***0***_ **)**	8.53 ± 5.37	7.98	3.56 - 11.35	***< 0.01***
**ACP (*T***_***1***_**)-to-MCP (*T***_***1***_)	3.87 ± 2.58	3.83	1.61–5.72	

ACP, Actual chin position; MCP, Modified chin point; T_0,_ Pre-surgery; T_1_, Post-surgery; mm, millimetres; SD, standard deviation; IQR, Interquartile range; *p < 0.01, considered statistically significant.

The results of the method validity assessment are presented in Table 4. An overall small systematic bias was observed between post-surgery ACP and post-surgery MCP for upper and lower lip positions in relation to ‘S’ and ‘E’ lines with a ME of −0.04 ± 1.27 (UL) and −0.10 ± 2.52 mm (LL), and −0.06 ± 1.67 mm (UL) and −0.11 ± 2.77 mm (LL) for ‘S’ and ‘E’ lines, respectively. The MAE was −0.04 ± 1.08 mm (UL) and −0.52 ± 1.98 mm (LL) for the ‘S’ line and 0.09 ± 1.60 mm (UL) and −0.87 ± 2.04 mm (LL) for the ‘E’ line, suggesting a small average disparity between the post-surgery ACP and post-surgery MCP.

**Table 4:** Method validity assessment.

	ACP* (T*_*1*_*) – *MCP* (T*_*1*_*)*					
	Mean error (mm)			Mean absolute error (mm)		
Variables	Mean ± SD	Minimum	Maximum	Mean ± SD	Minimum	Maximum
**UL**						
** S Line**	−0.04 ± 1.27	−2.90	2.32	−0.04 ± 1.08	−2.04	2.30
** E Line**	−0.06 ± 1.67	−3.72	3.04	0.09 ± 1.60	−3.04	3.72
**LL**						
** S Line**	−0.10 ± 2.52	−5.38	4.70	−0.52 ± 1.98	−5.38	3.15
** E Line**	−0.11 ± 2.77	−5.94	5.16	−0.87 ± 2.04	−5.20	4.57

ACP, Actual chin position; MCP, Modified chin point; T_1_, Post-surgery; UL, Upper Lip; LL, Lower Lip; mm, millimetres; SD, standard deviation.

**Fig 5:**
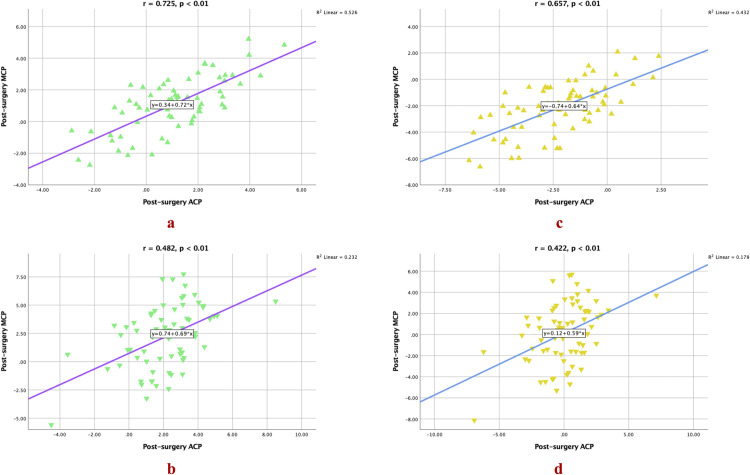
Visual depiction of the relationship between post-surgery ACP and post-surgery MCP. Lip positions for the ‘S’ line represented by green triangles and for the ‘E’ line represented by amber triangles. Upward-pointing triangles indicate UL positions, while downward-pointing triangles represent LL positions.

**Fig 6:**
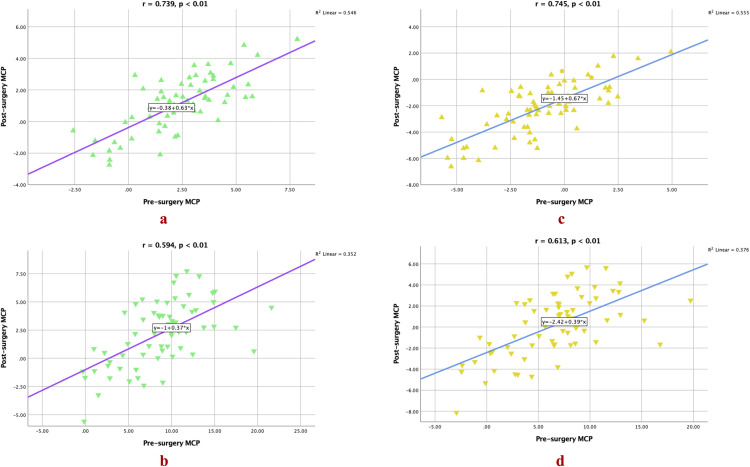
Visual illustration of the relationship between pre-surgery MCP and post-surgery MCP. Lip positions for the ‘S’ line represented by green triangles and for the ‘E’ line represented by amber triangles. Upward-pointing triangles indicate UL positions, while downward-pointing triangles represent LL positions.

Additionally, correlation analysis demonstrated a strong positive correlation between post-surgery ACP and post-surgery MCP (Fig 5a and 5c), as well as between pre-surgery MCP and post-surgery MCP (Fig 6a and 6c), for both ‘S’ and ‘E’ lines in the UL measurements. For the LL, a moderate positive correlation was observed between post-surgery ACP and post-surgery MCP (Fig 5b and 5d) for both ‘S’ and ‘E’ lines, as well as between pre-surgery MCP and post-surgery MCP (Fig 6b) for the ‘S’ line. Furthermore, the analysis also demonstrated a strong positive correlation between pre-surgery MCP and post-surgery MCP (Fig 6d) for the ‘E’ line. These findings provide evidence of the MCP’s higher accuracy and reliability as a predictor for the post-surgery profile.

MCP based post-surgery aesthetic lip positions for males and females are presented in Table 5. The results revealed that there was no statistically significant difference (*p* > 0.01) in the lip positions between males and females. However, males exhibited higher mean values of UL (1.17 ± 1.61 mm) and LL (2.95 ± 2.50 mm) relative to the ‘S’ line compared to females. When assessing lip positions relative to the ‘E’ line, females displayed higher mean values of UL (−2.14 ± 2.13 mm) compared to males, while males exhibited higher mean values of LL (0.85 ± 2.55 mm) compared to females. The majority of post-surgical lip positions (95% CI) in our cohort fell within ±2 mm of the S or E lines, consistent with the aesthetic threshold established in our previous work.

**Table 5:** Modified chin point based post-surgery aesthetic lip positions.

	Male		Female		
Variables	Mean ± SD (mm)	95% CI	Mean ± SD (mm)	95% CI	p*
**UL**					
** S Line**	1.17 ± 1.61	0.55 to 1.80	1.04 ± 1.80	0.46 to 1.62	> 0.01
** E Line**	−2.15 ± 1.78	−2.84 to −1.46	−2.14 ± 2.13	−2.82 to −1.46	
**LL**					
** S Line**	2.95 ± 2.50	1.98 to 3.91	1.63 ± 2.88	0.70 to 2.55	
** E Line**	0.85 ± 2.55	−0.14 to 1.84	−0.38 ± 3.05	−1.35 to 0.60	

MCP, Modified chin point; UL, Upper Lip; LL, Lower Lip; mm, millimetres; SD, standard deviation; CI, Confidence Interval; *p < 0.01, considered statistically significant

## Discussion

Successful orthognathic treatment aims to achieve functional and stable occlusion as well as enhanced facial aesthetics. To ensure that patients and surgeons have shared goals and expectations, it is imperative to establish a clear surgical plan. Within this framework, prediction of the post-surgery profile has become pivotal in the strategic planning of orthognathic surgery. It furnishes surgeons with valuable information to plan surgical manoeuvres and evaluate the necessity of orthodontic decompensation. By incorporating post-surgery profile determination into strategic planning, surgeons can enhance the precision and effectiveness of orthognathic treatment. Previous studies have determined the post-surgery profile using various techniques, including 2D methods like cephalometric tracing [39,40], and scanned images of cephalometric radiographs [41], as well as 3D approaches such as CT (Computed Tomography) volumes [42], CT/CBCT (Cone Beam Computed Tomography)-generated 3D mesh [43,44], and 3D scans [45]. However, the predictability of the post-surgery soft-tissue profile based on the chin point has not yet been investigated. Therefore, this study attempted to evaluate the validity of MCP as a reliable predictor for assessing the post-orthognathic surgery profile.

AI algorithms have found extensive application in the disciplines of OS and orthodontics for diagnosis and prediction [46–48]. When it comes to OS patients, where aesthetic concerns are paramount, determining the post-surgery profile becomes a crucial aspect of treatment planning. Such a prediction model can aid patients in assessing whether the proposed OS treatment aligns with their aesthetic expectations while also enabling orthognathic surgeons to optimize the treatment plan accordingly. However, predicting profile aesthetics is a complex process that relies on several factors [49], and no previously reported prediction model has exhibited sufficient reliability. Therefore, in this work, we developed AI-assisted automated in-house software specifically designed to determine the post-surgery profile. To the best of our knowledge, this is the first study to apply AI-assisted automated in-house software to examine the applicability of MCP as a valid predictor for post-surgery profiles. To validate the MCP, this study employed a methodological approach emphasizing automation and error minimization. Previous research has demonstrated that automated measurements offer faster evaluation and reduced variability [50]. Hence, specifically developed in-house software was employed in the present study to minimize human error and enhance the accuracy and reliability of determinations. To achieve these essential objectives, we employed an advanced deep learning model dubbed *BiSeNet*, which is specifically designed for semantic image segmentation tasks. It aims to accurately label each pixel in an image with the corresponding class or category, enabling precise object detection and segmentation. To further enhance the accuracy and performance of our AI-assisted in-house software, a training methodology called *Supervision by Registration* was utilized. It involves aligning or matching images or objects to a common coordinate system through a registration algorithm. This training technique helps the AI model better understand the spatial relationships between different objects or features in the data, leading to improved accuracy and robustness. Thus, the deployment of this AI-assisted automated in-house software ensured accuracy and reliability in the assessment of MCP.

Patients with clinically apparent facial asymmetry (soft tissue chin deviation > 3 mm) [51–53] often have a deviation of the pogonion to the right or left. The accurate location of the soft tissue chin point on 2D photographs and the identification of asymmetry based solely on these photographs could have posed challenges. A deviated pogonion could have influenced the accuracy of the UL and LL positions relative to the chin. To address this limitation, the present study utilized 3D facial images. These 3D images allowed for rotation, enabling the identification of a deviated chin. Moreover, 3D facial images provided high precision and accuracy [54,55]. The use of 3D facial images in the present analysis facilitated the identification of patients with asymmetry and their subsequent exclusion from the analysis, ensuring data homogeneity. This exclusion was essential to preserving the integrity and reliability of the findings.

Achieving desirable facial profile aesthetics requires a balanced lower third of the face. Previous studies have established the correlation between chin morphology, lip protrusion, and profile aesthetics [56,57]. Thus, in this study, lip positions were applied to assess the validity of the newly derived MCP as a reliable predictor. Unlike ACP, which may not accurately represent the desired post-surgical lip position due to variability in chin morphology, MCP provides a more stable and reproducible reference for predicting upper lip position, as demonstrated by our findings. The results strongly support using MCP as a reliable predictor for post-orthognathic surgery profile in Chinese OS patients. By comparing the positioning of the lips relative to the ACP and the MCP, the present study investigated the accuracy of the MCP in predicting the post-surgical lip position. The findings demonstrated that for the UL measurements, the SD values were generally smaller for the MCP (*T*_*0*_: S line, 2.00 mm; E line, 2.21 mm; *T*_*1*_: S line, 1.71 mm; E line, 1.98 mm) compared to the ACP (*T*_*0*_: S line, 2.78 mm; E line, 3.41 mm; *T*_*1*_: S line, 1.72 mm; E line, 2.05 mm) in both pre-surgery and post-surgery groups. This suggests that the positioning of the lips relative to the MCP was more consistent and reliable than the ACP for the UL measurements in both groups (Table 2). Rather than serving merely as a conversion metric between reference points, MCP establishes a new, more reliable standard for reference in surgical planning. However, for the LL measurements, the consistency and reliability were equivalent or slightly lower for the MCP compared to the ACP, as demonstrated by the higher SD values (Table 2). In light of this, a further assessment of the precision of the estimates and the consistency of lip positioning relative to the MCP was conducted (Table 2). A narrower CI was observed for the UL measurements relative to both ‘S’ and ‘E’ line variables for MCP [*T*_*0*_ (S line: UL, 1.84 to 2.81; E line: UL, −1.58 to −0.51); *T*_*1*_ (S line: UL, 0.68 to 1.51; E line: UL, −2.63 to −1.67)] compared to ACP [*T*_*0*_ (S line: UL, 0.29 to 1.63; E line: UL, −3.70 to −2.05); *T*_*1*_ (S line: UL, 0.63 to 1.47; E line: UL, −2.71 to −1.71)] in both pre- and post-surgery groups, suggesting that the MCP variables were more consistent and reliable than the ACP variables for the UL position concerning both the ‘S’ and ‘E’ lines. On the other hand, LL measurements exhibited somewhat wider CI values relative to ‘S’ and ‘E’ lines for MCP [*T*_*0*_ (S line: LL, 7.56 to 9.75; E line: LL, 5.40 to 7.59); *T*_*1*_ (S line: LL, 1.49 to 2.84; E line: LL, −0.57 to 0.83)] as compared to ACP [*T*_*0*_ (S line: LL, 5.34 to 6.70; E line: LL, 2.78 to 4.29); *T*_*1*_ (S line: LL, 1.60 to 2.54; E line: LL, −0.49 to 0.52)] in both pre- and post-surgery groups, thereby suggesting that the consistency and reliability for the LL position were similar or slightly lower for the MCP compared to the ACP. Overall, the narrower CI values for MCP variables compared to ACP variables in both pre-surgery and post-surgery groups suggest greater consistency and reliability in lip positioning relative to MCP, specifically for the UL position with respect to both the ‘S’ and ‘E’ lines. This consistency is critical for virtual surgical planning (VSP), where precise reference points are essential for simulating post-surgical outcomes. By minimizing variability in UL positioning, MCP enables surgeons to predict aesthetic results with greater confidence, reducing intraoperative adjustments and improving alignment between planned and actual outcomes. By adopting MCP as the reference point, surgeons can achieve more predictable and aesthetically pleasing results in VSP, thereby improving patient satisfaction and surgical precision. Although, the CI values for the LL measurements in the MCP group indicate a modestly lower consistency in lip positioning relative to the ACP group, the overall data demonstrate that the MCP offers greater precision and consistency. Additionally, a comparative analysis of chin position differences before and after surgery, relative to the ACP and MCP, revealed a substantial reduction in the ACP-to-MCP distance in the post-surgery group (3.83 mm) compared to the pre-surgery group (7.98 mm) (Table 3). This highlights the significant impact of orthognathic surgery on lip positioning relative to the chin (Fig 4). Based on these findings, MCP shows promise as a reliable predictor for post-surgery profiles in OS patients.

Accurate prediction is crucial for evaluating treatment feasibility, optimizing case management, and improving patient understanding and acceptance of the recommended treatment [58]. As such, in the present study, the method validity was assessed in terms of ME and MAE. Our analysis revealed a small systematic bias and a minor average disparity between the post-surgery ACP and MCP for both UL and LL positions. The ME and MAE values for the present prediction model were much smaller compared to the ME and MEA values reported in previous studies [56,59]. For the UL measurements, the ME and MAE were relatively small with low SD values (ME: S, 1.27 mm; E, 1.67 mm; MAE: S, 1.08 mm; E, 1.60 mm; Table 4), indicating minimal bias and high accuracy in MCP-based predictions of post-surgery UL position. This level of error—where both ME and MAE are below 2 mm—suggests that the MCP provides clinically reliable estimations, ensuring that planned and actual outcomes align closely. Such precision is critical for VSP and patient consultations, as it allows surgeons to set realistic expectations and improve acceptance of treatment plans through accurate visual simulations. This enhanced predictability directly translates to improved patient outcomes, as surgeons can more confidently plan and execute procedures that meet both functional and aesthetic goals. Furthermore, the strong positive correlation observed between post-surgery ACP and post-surgery MCP, as well as between pre-surgery MCP and post-surgery MCP for both ‘S’ line and ‘E’ line measurements (Figs 5 and 6), further supports the consistency and reliability of MCP in estimating the post-surgery UL position. On the other hand, for the LL measures, the ME and MAE were slightly larger compared to the UL measurements, with wider ranges (as indicated by the minimum and maximum values) and slightly higher SD values (Table 4). However, there was still moderate positive correlation observed between post-surgery ACP and post-surgery MCP, as well as between pre-surgery MCP and post-surgery MCP for both ‘S’ line and ‘E’ line measurements (Figs 5 and 6), which was in agreement with *Lu et al.’s* findings [59] who also reported a moderate correlation between lip changes and chin point. This indicates that the MCP can still be a moderately reliable in estimating the post-surgery LL position. In contrast to the findings of previous studies that reported variable predictability for the UL and LL positions [60–63], the validity of MCP as a reliable predictor for the post-surgery profile in the present analysis can be justified, especially for the UL. This is supported by the small ME and MAE, strong positive correlations, and moderate positive correlations observed in the present analysis. While p-values >0.01 for LL measurements indicate that observed differences may fall within random variation, the small ME and MAE (<3 mm) and moderate correlations suggest MCP remains a clinically useful reference for LL positioning. For UL, the strong correlations and minimal bias (<1.7 mm) justify MCP’s adoption in virtual surgical planning, even when strict statistical thresholds are unmet. Thus, based on the findings of the present study, MCP can be considered a reliable predictor for the post-surgery profile in this population.

In the pursuit of establishing MCP-based post-surgery aesthetic lip positions, the current study analysed the UL and LL lip positions relative to ‘S’ and ‘E’ lines in both males and females (Table 5). The results showed that in both the genders, UL position was slightly ahead (males, 0.55 to 1.80 mm; females, 0.46 to 1.62 mm), while the LL position was ahead of the ‘S’ line (males, 1.98 to 3.91 mm; females, 0.70 to 2.55 mm), at 95% CI, thus suggesting that post-surgery, a slightly protrusive UL position and a protrusive LL position relative to the ‘S’ line would be more aesthetic in both genders. However, when assessing the lip positions in relation to the ‘E’ line, UL was found to be behind the ‘E’ line in both genders (males, −2.84 to −1.46 mm; females, −2.82 to −1.46 mm), at 95% CI. In addition, LL in males was ‘on or slightly ahead’ (−0.14 to 1.84 mm), while in females it was ‘on or slightly behind’ the ‘E’ line (−1.35 to 0.60 mm), considering a 95% CI. These positional trends indicate that post-surgery, a retrusive UL position in both males and females and ‘on the E line’ or slightly protrusive LL position in males, while ‘on the E line’ or slightly retrusive LL position in females could serve as preliminary guidelines for aesthetic planning. However, this study did not directly evaluate patient or clinician perceptions of facial aesthetics. Therefore, the relationship between these positional trends and perceived attractiveness remains to be validated in future studies incorporating subjective assessments.

In terms of magnitude, we suggest that during pre-surgical planning, the UL position can be planned approximately 0.5–0.6 mm (95% CI rounded to the nearest decibel) ahead of the ‘S’ line in both genders. Similarly, the LL position may be planned approximately 2 mm ahead relative to the ‘S’ line in males and 0.7 mm ahead in females. For the ‘E’ line, planning the UL position about 2.8 mm behind in both genders, and the LL position between 0.1 mm behind to 1.8 mm ahead in males and 1.4 mm behind to 0.6 mm ahead in females, may be optimal. These recommendations for pre-surgical lip positioning are based on the ± 2 mm threshold previously validated as aesthetically optimal in Chinese profiles [23]. By using MCP, we enable the application of this evidence-based criterion to Class II and III skeletal patterns, which were previously not addressable with S and E lines alone. These recommendations, grounded in both our findings and previous validation, may contribute to enhanced aesthetic outcomes in orthognathic surgery patients. These findings suggest that, for Chinese OS patients, the MCP could help predict post-surgery lip positions, and its incorporation in pre-operative treatment planning may improve alignment between planned and actual outcomes.

In the present study, we also noticed some interesting findings. Despite the UL position being behind the ‘E’ line as advocated by *Ricketts et al.*, the mean absolute values for both males and females were smaller than the range described by *Ricketts et al*. Moreover, both the UL and LL positions were found to be ahead of the ‘S’ line, contradicting the criteria proposed by *Steiner et al*. These contrasting results can be attributed to ethnic disparities, as normative values specific to one ethnic group may not necessarily apply to other ethnicities [64]. It is worth noting that the subjects in the studies conducted by *Ricketts et al*. and *Steiner et al.* were predominantly Caucasians, while our study focused on Chinese subjects, who generally have more protrusive lips than Caucasians. This ethnic difference likely contributes to the varying results. Furthermore, regardless of the reference line (S or E), both genders displayed a more protrusive LL (S line: males, 2.95 mm; females, 1.63 mm; E line: males, 0.85 mm; females, −0.38 mm) compared to UL (S line: males, 1.17 mm; females, 1.04 mm; E line: males, −2.15 mm; females, −2.14 mm). This finding aligns with the previous studies [23,65] and can be attributed to the normal labial inclination of mandibular incisors in Chinese subjects [66].

**Fig 7:**
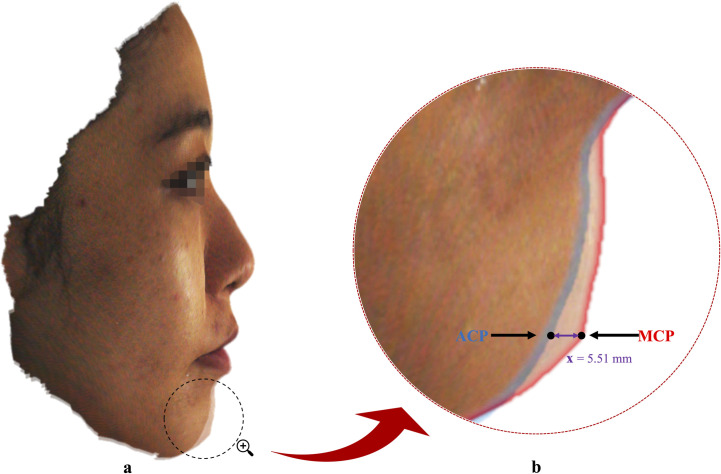
A visual representation of the registered post-surgery ACP and MCP images highlighting the difference in post-surgery profiles based on ACP and MCP. The blue outline represents the post-surgery profile based on ACP, while the red outline represents the predicted post-surgery profile based on MCP. The difference between the ACP-based post-surgery profile and the MCP-based predicted post-surgery profile can be easily observed. Note that the horizontal distance between ACP and MCP (denoted by ‘x’) is specific to this patient.

**Fig 8:**
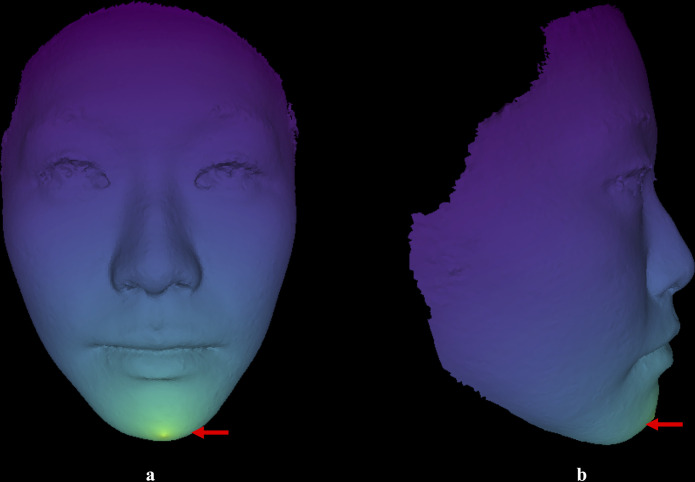
Colour-mapped image of the superimposed post-surgery ACP and MCP images. The yellow region (indicated by a red arrow) demonstrates the difference between ACP and MCP, confirming that the post-surgery profile based on ACP may not be similar to the post-surgery profile based on MCP.

The methodology employed in the present study offers several advantages. Firstly, the proposed program is fully automated and only requires a textured facial scan as input. This allows for easy and efficient processing of the scan and the computation of necessary metrics. Additionally, the program is easily expandable, allowing for the calculation of various metrics using parameterization. It is also adaptable to a wide range of inputs, including smartphone-generated facial scans, eliminating the need for expensive professional facial scanners. However, it is crucial to ensure that the input facial scan is well-captured, accurately capturing all facial features inclusively. Besides, the MCP-based prediction model used in our study is a quick, practical, and relatively accurate method for determining post-surgery profile in patients with skeletal class I, II, or III deformities. In terms of clinical applicability, it can be easily incorporated into pre-surgical planning, enabling precise determination of the desired post-surgical lip position. Additionally, the MCP based post-surgery aesthetic lip positions presented in this study can provide guidance on the degree of maxillomandibular advancement or setback, leading to an aesthetic post-surgery profile. This makes MCP a valuable tool for predicting and enhancing the overall aesthetic outcome of OS (Fig 7). The findings of this study have important implications for orthognathic surgeons and orthodontists. By replacing ACP with MCP in VSP, clinicians can leverage a more reliable reference point to simulate post-surgical lip positions. For example, the narrower CI for MCP-based UL measurements (Table 2) indicate that surgeons can plan maxillomandibular advancements or setbacks with greater precision, minimizing the risk of over- or under-correction. This directly enhances patient satisfaction by aligning surgical outcomes with preoperative simulations. Our study also serves as a reference for clinicians and patients, providing insight into the expected treatment outcome before commencing orthognathic treatment. By introducing MCP as a novel reference point in preoperative planning, our findings lay the groundwork for reducing reliance on subjective assessments and improving the reproducibility of aesthetic outcomes. As virtual surgical workflows and AI-driven planning tools continue to evolve, future integration of MCP-based prediction models may further enhance the efficiency and accuracy of orthognathic surgical planning. In addition, this study contributes to the existing literature by emphasizing the significance of considering the chin position when predicting post-surgical lip aesthetics. The traditional approach of relying solely on ACP may not accurately represent the desired lip position after orthognathic surgery. By introducing the MCP as an additional reference point, we establish a more comprehensive framework for evaluating and predicting the post-surgical profile (Fig 8). Future studies should focus on the development and validation of additional metrics using parameterization to enhance the accuracy and reliability of the proposed prediction model. Specifically, comparative studies in African and European populations are needed to determine whether the consistency of MCP observed here is universal or influenced by ethnic variations in facial convexity. Such research will clarify whether MCP requires population-specific adjustments or can serve as a global standard for orthognathic planning. Furthermore, the MCP-based prediction model can be compared with other existing models, providing a comprehensive understanding of their strengths and weaknesses. Such comparison will assist orthognathic surgeons and orthodontists in selecting the most appropriate prediction model, ultimately leading to improved treatment outcomes. In summary, this study demonstrates that MCP is a superior reference point for predicting post-surgical lip position, particularly for the upper lip, in Chinese OS patients. Its adoption in virtual surgical planning can enhance both the precision of surgical simulations and the reproducibility of aesthetic outcomes, marking a significant advancement in orthognathic surgery planning.

Despite conducting a comprehensive analysis, our study had certain limitations. Firstly, the parameterization was only approximate, which may have resulted in some imprecision. The simplifications and approximations adopted during parameterization could have caused a loss of detail or distortion, potentially affecting the measurement accuracy. Secondly, highly variable factors such as the thickness, tonicity, posture, and length of the soft tissue covering were not investigated, which could have influenced the prediction accuracy. Further, it should be noted that our study only focused on a specific patient population (OS patients) and included only Chinese subjects. Therefore, the applicability of our findings to other ethnic groups and populations, such as those with facial asymmetry, may be limited. Facial morphology and aesthetic norms can differ significantly across ethnic groups such as Africans and Europeans, and the angle of facial convexity used to derive MCP may require population-specific calibration. Thus, our results may not directly apply to other populations. Future research should validate the MCP approach in diverse cohorts and integrate ethnic-specific soft-tissue data into AI-driven planning tools to assess generalizability. Furthermore, validation of the MCP across diverse populations is necessary to establish the generalizability of our findings. Such investigations would help establish whether the trends observed in our study are universally applicable or vary due to population-specific anatomical differences. Additionally, while our recommendations for lip positioning are grounded in validated aesthetic criteria from our previous work, no direct evaluation of patient- or clinician-rated esthetic outcomes was performed in this study. Future research should include subjective assessments to confirm whether the proposed lip positions correspond to improved perceived facial attractiveness.

## Conclusions

The present study provides evidence supporting the effectiveness of MCP in guiding aesthetic lip positioning in OS patients, as demonstrated by comparisons of lip positioning relative to ACP and MCP. The study draws the following specific conclusions:

1. The UL positioning relative to the MCP was generally more consistent and reliable than ACP, but for the LL measurements, the consistency and reliability were equivalent or slightly lower for the MCP than ACP. Nonetheless, the MCP variables remained more precise and consistent overall than the ACP variables.
2. Orthognathic surgery had a significant impact on the positioning of the lips relative to the chin, as demonstrated by a significant reduction in the ACP-to-MCP distance in the post-surgery group compared to the pre-surgery group.
3. The MCP was consistent and reliable in estimating the post-surgery UL position as well as moderately reliable in estimating the post-surgery LL position. Overall, based on small ME and MAE values along with strong positive correlations and moderate positive correlations, MCP can be considered a reliable predictor for the post-surgery profile.
4. Based on the observed post-surgical lip positions in our cohort, we propose that planning for a slightly protrusive UL position and a protrusive LL position relative to the ‘S’ line in both males and females and a retrusive UL position in both males and females and ‘on the E line’ or slightly protrusive LL position in males while ‘on the E line’ or slightly retrusive LL position in females may contribute to improved aesthetic outcomes.

In conclusion, this study validates the use of the MCP as a reliable predictor for post-orthognathic surgery profile. The practicality and potential benefits of incorporating the MCP into pre-surgical planning make it a valuable tool for orthognathic surgeons.

## References

[1] Faure JC, Rieffe C, Maltha JC. The influence of different facial components on facial aesthetics. Eur J Orthod. 2002;24(1):1–7. doi: 10.1093/ejo/24.1.1 11887373

[2] Liu BC-L, Lee I-C, Lo L-J, Ko EW-C. Investigate the oral health impact and quality of life on patients with malocclusion of different treatment needs. Biomed J. 2019;42(6):422–9. doi: 10.1016/j.bj.2019.05.009 31948607 PMC6962747

[3] Jung M-H. Evaluation of the effects of malocclusion and orthodontic treatment on self-esteem in an adolescent population. Am J Orthod Dentofacial Orthop. 2010;138(2):160–6. doi: 10.1016/j.ajodo.2008.08.040 20691357

[4] Cunningham MR, Barbee AP, Pike CL. What do women want? Facialmetric assessment of multiple motives in the perception of male facial physical attractiveness. J Pers Soc Psychol. 1990;59(1):61–72. doi: 10.1037//0022-3514.59.1.61 2213490

[5] Ghorbanyjavadpour F, Rakhshan V. Factors associated with the beauty of soft-tissue profile. Am J Orthod Dentofacial Orthop. 2019;155(6):832–43. doi: 10.1016/j.ajodo.2018.07.020 31153504

[6] Schendel SA. Anthropometry of the head and face. Plast Reconst Surg. 1995;96(2):480.

[7] Giddon DB. Orthodontic applications of psychological and perceptual studies of facial esthetics. Semin Orthod. 1995;1(2):82–93. doi: 10.1016/s1073-8746(95)80095-6 8935047

[8] Furquim BA, de Freitas KM, Janson G, Simoneti LF, de Freitas MR, de Freitas DS. Class III malocclusion surgical-orthodontic treatment. Case Rep Dent. 2014;2014:868390. doi: 10.1155/2014/868390 25431691 PMC4241289

[9] Nanda RS, Ghosh J. Facial soft tissue harmony and growth in orthodontic treatment. Semin Orthod. 1995;1(2):67–81. doi: 10.1016/s1073-8746(95)80094-8 8935046

[10] Proffit WRFH, Larson BE, Sarver DM. Contemporary orthodontics. 6th ed. 2019.

[11] Reyneke JP, Ferretti C. Diagnosis and planning in orthognathic surgery. In: Bonanthaya K, Panneerselvam E, Manuel S, Kumar VV, Rai A, editors. Oral and maxillofacial surgery for the clinician. Singapore: Springer Singapore; 2021. pp. 1437–62.

[12] Ren H, Chen X, Zhang Y. Correlation between facial attractiveness and facial components assessed by laypersons and orthodontists. J Dent Sci. 2021;16(1):431–6. doi: 10.1016/j.jds.2020.07.012 33384831 PMC7770325

[13] Naini FB, Garagiola U, Wertheim D. Analysing chin prominence in relation to the lower lip: The lower lip-chin prominence angle. J Craniomaxillofac Surg. 2019;47(8):1310–6. doi: 10.1016/j.jcms.2019.06.002 31331858

[14] Eppley BL. Chin reshaping in profileplasty: augmentative and reductive strategies. Facial Plast Surg. 2019;35(5):499–515. 31639875 10.1055/s-0039-1695750

[15] Naini FB, Donaldson ANA, McDonald F, Cobourne MT. Assessing the influence of lower facial profile convexity on perceived attractiveness in the orthognathic patient, clinician, and layperson. Oral Surg Oral Med Oral Pathol Oral Radiol. 2012;114(3):303–11. doi: 10.1016/j.tripleo.2011.07.031 22883980

[16] de Sousa Filho JL, Rodrigues da Silva AMB, Trivellato AE, Rodrigues da Silva MA, Sverzut CE. Evaluation of chin morphology after two-jaw orthognathic surgery: a retrospective study using stereophotogrammetry. J Oral Res Rev 2022;14(1):1–6. doi: 10.4103/jorr.jorr_42_21

[17] Chen HL, Yueh-Tse L, Wang Y-C. Profile angle defines soft tissue chin position in adults. Taiwan J Orthod. 2014;26(1):1–13.

[18] Lee EI. Aesthetic alteration of the chin. Semin Plast Surg. 2013;27(3):155–60. doi: 10.1055/s-0033-1357113 24872762 PMC3805726

[19] González-Ulloa M, Stevens E. The role of chin correction in profileplasty. Plast Reconstr Surg. 1968;41(5):477–86. doi: 10.1097/00006534-196805000-00010 5652638

[20] Ricketts RM. Planning treatment on the basis of the facial pattern and an estimate of its growth. Angle Orthod. 1957;27(1):14–37.

[21] Steiner CC. Cephalometrics for you and me. Am J Orthod. 1953;39(10):729–55.

[22] Merrifield LL. The profile line as an aid in critically evaluating facial esthetics. Am J Orthod. 1966;52(11):804–22. doi: 10.1016/0002-9416(66)90250-8 5223046

[23] Ng JHH, Singh P, Wang Z, Yang Y, Khambay BS, Gu M. The reliability of analytical reference lines for determining esthetically pleasing lip position: An assessment of consistency, sensitivity, and specificity. Am J Orthod Dentofacial Orthop. 2023;164(1):e14–26. doi: 10.1016/j.ajodo.2023.04.011 37227323

[24] Arnett GW, Bergman RT. Facial keys to orthodontic diagnosis and treatment planning. Part I. Am J Orthod Dentofacial Orthop. 1993;103(4):299–312. doi: 10.1016/0889-5406(93)70010-L 8480695

[25] Arnett GW, Bergman RT. Facial keys to orthodontic diagnosis and treatment planning--Part II. Am J Orthod Dentofacial Orthop. 1993;103(5):395–411. doi: 10.1016/s0889-5406(05)81791-3 8480709

[26] Anić-Milosević S, Lapter-Varga M, Slaj M. Analysis of the soft tissue facial profile by means of angular measurements. Eur J Orthod. 2008;30(2):135–40. doi: 10.1093/ejo/cjm116 18263886

[27] Resnick CM, Kim S, Yorlets RR, Calabrese CE, Peacock ZS, Kaban LB. Evaluation of Andrews’ analysis as a predictor of ideal sagittal maxillary positioning in orthognathic surgery. J Oral Maxillofac Surg. 2018;76(10):2169–76. doi: 10.1016/j.joms.2018.03.013 29654777

[28] Cheng M, Zhang X, Wang J, Yang Y, Li M, Zhao H, et al. Prediction of orthognathic surgery plan from 3D cephalometric analysis via deep learning. BMC Oral Health. 2023;23(1):161. doi: 10.1186/s12903-023-02844-z 36934241 PMC10024836

[29] Kato RM, Parizotto JDOL, Oliveira PHJD, Gonçalves JR. Artificial intelligence in orthognathic surgery – a narrative review of surgical digital tools and 3D orthognathic surgical planning. J Calif Dent Assoc. 2023;51(1):2202444.

[30] Kim YH, Kim I, Kim Y-J, Kim M, Cho J-H, Hong M, et al. The prediction of sagittal chin point relapse following two-jaw surgery using machine learning. Sci Rep. 2023;13(1):17005. doi: 10.1038/s41598-023-44207-2 37813915 PMC10562368

[31] Aljabaa AH. Lateral cephalometric analysis of the nasal morphology among Saudi adults. Clin Cosmet Investig Dent. 2019;11:9–17. 10.2147/CCIDE.S190230 PMC6338237 30679927

[32] Athanasiou AE. Orthodontic cephalometry. 1st ed. Maryland Heights, MI, USA: Mosby-Wolfe; 1995.

[33] Tseng Y-C, Yang C, Cheng J-H, Pan C-Y, Chou S-T, Chen C-M. Improvement in lip appearance (lateral and frontal aspects) following mandibular setback surgery. J Stomatol Oral Maxillofac Surg. 2019;120(4):317–21. doi: 10.1016/j.jormas.2019.02.010 30794882

[34] Jakobsone G, Vuollo V, Pirttiniemi P. Reproducibility of Natural Head Position assessed with stereophotogrammetry. Orthod Craniofac Res. 2020;23(1):66–71. doi: 10.1111/ocr.12344 31514260

[35] Yu C, Wang J, Peng C, Gao C, Yu G, Sang N. BiSeNet: Bilateral Segmentation Network for Real-Time Semantic Segmentation. Cham: Springer International Publishing; 2018.

[36] Han H, Zhu L, Singh P r a d e e p, Hsung RTC, Leung YY, Komura T, et al. Facial surgery preview based on the orthognathic treatment prediction. Comput Methods Programs Biomed. 2024;241:11045. doi: 10.1016/j.cmpb.2024.11045 40286420

[37] Dong X, Yu S-I, Weng X, Wei S-E, Yang Y, Sheikh Y. Supervision-by-Registration: An Unsupervised Approach to Improve the Precision of Facial Landmark Detectors. 2018 IEEE/CVF Conference on Computer Vision and Pattern Recognition (CVPR): IEEE Computer Society; 2018. pp. 360–8.

[38] Sagonas C, Tzimiropoulos G, Zafeiriou S, Pantic M. 300 Faces in-the-Wild Challenge: The First Facial Landmark Localization Challenge. 2013 IEEE International Conference on Computer Vision Workshops. 2013.

[39] Peterman RJ, Jiang S, Johe R, Mukherjee PM. Accuracy of Dolphin visual treatment objective (VTO) prediction software on class III patients treated with maxillary advancement and mandibular setback. Prog Orthod. 2016;17(1):19. doi: 10.1186/s40510-016-0132-2 27312722 PMC4911347

[40] de Lira ADLS, de Moura WL, Artese F, Bittencourt MAV, Nojima LI. Surgical prediction of skeletal and soft tissue changes in treatment of Class II. J Craniomaxillofac Surg. 2013;41(3):198–203. doi: 10.1016/j.jcms.2012.07.009 23201327

[41] Pektas ZO, Kircelli BH, Cilasun U, Uckan S. The accuracy of computer-assisted surgical planning in soft tissue prediction following orthognathic surgery. Int J Med Robot. 2007;3:64–71. doi: 10.1002/rcs.127 17441028

[42] Resnick CM, Dang RR, Glick SJ, Padwa BL. Accuracy of three-dimensional soft tissue prediction for Le Fort I osteotomy using Dolphin 3D software: a pilot study. Int J Oral Maxillofac Surg. 2017;46(3):289–95. doi: 10.1016/j.ijom.2016.10.016 27856149

[43] Shafi MI, Ayoub A, Ju X, Khambay B. The accuracy of three-dimensional prediction planning for the surgical correction of facial deformities using Maxilim. Int J Oral Maxillofac Surg. 2013;42(7):801–6. doi: 10.1016/j.ijom.2013.01.015 23465803

[44] Terzic A, Combescure C, Scolozzi P. Accuracy of computational soft tissue predictions in orthognathic surgery from three-dimensional photographs 6 months after completion of surgery: a preliminary study of 13 patients. Aesthetic Plast Surg. 2014;38(1):184–91. doi: 10.1007/s00266-013-0248-4 24337148

[45] Liebregts JHF, Timmermans M, De Koning MJJ, Bergé SJ, Maal TJJ. Three-dimensional facial simulation in bilateral sagittal split osteotomy: a validation study of 100 patients. J Oral Maxillofac Surg. 2015;73(5):961–70. doi: 10.1016/j.joms.2014.11.006 25795178

[46] Li P, Kong D, Tang T, Su D, Yang P, Wang H. Orthodontic treatment planning based on artificial neural networks. Sci Rep. 2019;9(1):2037. 30765756 10.1038/s41598-018-38439-w PMC6375961

[47] Franks SL, Bakshi A, Khambay BS. The validity of using profile predictions for class III patients planned for bimaxillary orthognathic surgery. Br J Oral Maxillofac Surg. 2022;60(4):507–12. doi: 10.1016/j.bjoms.2021.09.016 35346522

[48] Rupperti S, Winterhalder P, Krennmair S, Holberg S, Holberg C, Mast G, et al. Changes in the facial soft tissue profile after maxillary orthognathic surgery. J Orofac Orthop. 2022;83(3):215–20. doi: 10.1007/s00056-021-00294-2 33881549 PMC9038810

[49] Maetevorakul S, Viteporn S. Factors influencing soft tissue profile changes following orthodontic treatment in patients with Class II Division 1 malocclusion. Prog Orthod. 2016;17:13. doi: 10.1186/s40510-016-0125-1 27135067 PMC4852168

[50] Freire CMV, Ribeiro ALP, Barbosa FBL, Nogueira AI, de Almeida MCC, Barbosa MM, et al. Comparison between automated and manual measurements of carotid intima-media thickness in clinical practice. Vasc Health Risk Manag. 2009;5:811–7. 19812693 PMC2754094

[51] Ajmera DH, Hsung RT, Singh P, Wong NSM, Yeung AWK, Lam WYH, et al. Three-dimensional assessment of facial asymmetry in Class III subjects. Part 1: a retrospective study evaluating postsurgical outcomes. Clin Oral Investig. 2022;26(7):4947–66. doi: 10.1007/s00784-022-04463-4 35320382 PMC9276556

[52] Ajmera DH, Zhang C, Ng JHH, Hsung RT-C, Lam WYH, Wang W, et al. Three-dimensional assessment of facial asymmetry in class III subjects, part 2: evaluating asymmetry index and asymmetry scores. Clin Oral Investig. 2023;27(10):5813–26. doi: 10.1007/s00784-023-05193-x 37615775 PMC10560190

[53] Ajmera DH, Singh P, Leung YY, Khambay BS, Gu M. Establishment of the mid-sagittal reference plane for three-dimensional assessment of facial asymmetry: a systematic review : Establishment of the mid-sagittal reference plane: a systematic review. Clin Oral Investig. 2024;28(4):242. doi: 10.1007/s00784-024-05620-7 38575839 PMC10995046

[54] Gašparović B, Morelato L, Lenac K, Mauša G, Zhurov A, Katić V. Comparing Direct Measurements and Three-Dimensional (3D) Scans for Evaluating Facial Soft Tissue. Sensors (Basel). 2023;23(5):2412. doi: 10.3390/s23052412 36904614 PMC10007047

[55] Singh P, Hsung RT-C, Ajmera DH, Leung YY, McGrath C, Gu M. Can smartphones be used for routine dental clinical application? A validation study for using smartphone-generated 3D facial images. J Dent. 2023;139:104775. doi: 10.1016/j.jdent.2023.104775 37944629

[56] Guo R, Tian Y, Li X, Li W, He D, Sun Y. Facial profile evaluation and prediction of skeletal class II patients during camouflage extraction treatment: a pilot study. Head Face Med. 2023;19(1):51. doi: 10.1186/s13005-023-00397-8 38044428 PMC10694895

[57] Huang Y-P, Li W. Correlation between objective and subjective evaluation of profile in bimaxillary protrusion patients after orthodontic treatment. Angle Orthod. 2015;85(4):690–8. doi: 10.2319/070714-476.1 25347046 PMC8611736

[58] Kolokitha OE. Validity of a manual soft tissue profile prediction method following mandibular setback osteotomy. Eur J Dent. 2007;1(4):202–11. 19212468 PMC2609908

[59] Lu W, Song G, Sun Q, Peng L, Zhang Y, Wei Y, et al. Analysis of facial features and prediction of lip position in skeletal class III malocclusion adult patients undergoing surgical-orthodontic treatment. Clin Oral Investig. 2021;25(9):5227–38. doi: 10.1007/s00784-021-03830-x 33590299

[60] Joss CU, Joss-Vassalli IM, Bergé SJ, Kuijpers-Jagtman AM. Soft tissue profile changes after bilateral sagittal split osteotomy for mandibular setback: a systematic review. J Oral Maxillofac Surg. 2010;68(11):2792–801. doi: 10.1016/j.joms.2010.04.020 20708321

[61] Kaipatur NR, Flores-Mir C. Accuracy of computer programs in predicting orthognathic surgery soft tissue response. J Oral Maxillofac Surg. 2009;67(4):751–9. doi: 10.1016/j.joms.2008.11.006 19304030

[62] Joss CU, Joss-Vassalli IM, Kiliaridis S, Kuijpers-Jagtman AM. Soft tissue profile changes after bilateral sagittal split osteotomy for mandibular advancement: a systematic review. J Oral Maxillofac Surg. 2010;68(6):1260–9. doi: 10.1016/j.joms.2010.01.005 20381940

[63] Donatsky O, Bjørn-Jørgensen J, Hermund NU, Nielsen H, Holmqvist-Larsen M, Nerder PH. Immediate postoperative outcome of orthognathic surgical planning, and prediction of positional changes in hard and soft tissue, independently of the extent and direction of the surgical corrections required. Br J Oral Maxillofac Surg. 2011;49(5):386–91. doi: 10.1016/j.bjoms.2010.06.005 20621403

[64] Nongthombam H, Kumar M, Goyal M, Abrar M, Shaha KS, Kumar S. Regional influence on the aesthetic preference of different Mongolian profiles: A comparative study of assessors from Northeast and Mainland India. Int Orthod. 2023;21(2):100730. doi: 10.1016/j.ortho.2023.100730 36773557

[65] Erbay EF, Caniklioğlu CM, Erbay SK. Soft tissue profile in Anatolian Turkish adults: Part I. Evaluation of horizontal lip position using different soft tissue analyses. Am J Orthod Dentofacial Orthop. 2002;121(1):57–64. doi: 10.1067/mod.2002.119780 11786873

[66] Gu Y, McNamara JA, Sigler LM, Baccetti T. Comparison of craniofacial characteristics of typical Chinese and Caucasian young adults. Eur J Orthod. 2011;33(2):205–11. doi: 10.1093/ejo/cjq054 20709723

